# Heart Rate Variability Mediates the Association Between Fear of Pain and Pain Perception: An Exploratory Study in Healthy Controls

**DOI:** 10.3390/jcm15051705

**Published:** 2026-02-24

**Authors:** Alessandra Venezia, Harriet Fawsitt-Jones, Elena Makovac

**Affiliations:** 1 Department of Neuroimaging, Institute of Psychology, Psychiatry & Neuroscience, King’s College London, London SE5 8AF, UK; alessandra.venezia@kcl.ac.uk (A.V.); harriet.fawsitt-jones@kcl.ac.uk (H.F.-J.); 2 Department of Life Sciences, Division of Psychology, Brunel University London, London UB8 3PH, UK

**Keywords:** cold pain, heart rate variability, pain, fear of pain, pain catastrophising

## Abstract

**Background/Objectives**: Fear of pain (FoP) is a critical psychological factor influencing the experience of pain, yet the mechanisms behind this relationship remain unclear. HRV, indexed here by resting RMSSD, reflects individual differences in cardiac vagal tone and has been linked to pain perception and pain-related psychological processes. In this exploratory, cross-sectional study, we examined whether HRV mediates the relationship between FoP and the subjective perception of pain intensity. **Methods**: Twenty-two healthy participants completed several self-reported measures, including the Fear of Pain Questionnaire (FPQ-SF), and underwent an experimental cold pain induction, as well as a continuous recording of HR at rest. Mediation analysis was performed to assess the indirect effect of HRV, calculated via the Root Mean Square Successive Difference (RMSSD), on cold pain perception. **Results**: In correlational analyses, the Fear of Severe Pain (FoSP) subscale was associated with lower resting logRMSSD and higher cold pain ratings. In mediation models, the pattern of results was consistent with an indirect association between FoSP and cold pain ratings via logRMSSD (bootstrap 90% CI), while the direct path from FoSP to pain was not significant. **Conclusions**: These preliminary findings are hypothesis-generating and suggest that individual differences in resting HRV may be one physiological correlate of the fear–pain relationship in healthy controls, rather than an index of autonomic responses during pain itself. Larger longitudinal and experimental studies are needed to test temporal ordering, specificity across FoP components, and whether autonomic measures during pain better explain fear–pain coupling.

## 1. Introduction

Fear of pain (FoP) describes the anticipation or fear of experiencing pain, leading to pain avoidance [1], as well as increased pain sensitivity and perceived pain intensity. This fear emerges after painful incidents and plays a critical role in shaping future pain perception. Catastrophizing and subsequent pain-related fear are central to “fear-avoidance” models of chronic pain [2,3], and a substantial body of evidence links these processes to disability and reduced physical functioning in individuals with chronic pain [4].

Despite strong theoretical models, the mechanisms linking FoP to perceived pain intensity remain incompletely understood. While individuals with heightened FoP tend to report more adverse pain experiences [5], meta-analytic evidence suggests that the magnitude of this association is small to moderate [6]. Moreover, some studies report weak or inconsistent prediction of pain perception by FoP and catastrophising [7], indicating that additional psychological or physiological mechanisms may account for unexplained variance. FoP induces hypervigilance towards pain [8], which in turn could influence the perceived pain intensity via top-down facilitation [9], and attentional biases towards pain-related information [10]. In addition, contextual and expectancy-related factors may further shape subjective pain reports independently of physiological mechanisms. Experimental evidence demonstrates that verbal suggestion and instructional framing can significantly modulate pain perception even in the absence of changes in nociceptive input [11], highlighting the potential contribution of non-specific contextual effects to variability in pain ratings.

The autonomic nervous system (ANS) plays a central role in our perception of both fear and pain. Heart rate variability (HRV), which measures the time variations between consecutive heartbeats, serves as an al indicator of the ANS’s ability to dynamically regulate homeostasis [12]. Lower HRV is an index of reduced psychological and physiological adaptability, linked to poorer emotional regulation and adverse health outcomes. In contrast, higher HRV is associated with greater emotion regulation capacities and stress resilience [13].

HRV is known to be reduced in individuals with chronic pain [14,15,16], and this reduction is often associated with pain-related catastrophising and depressive symptoms [17]. While a substantial body of research has shown that fear and anticipatory anxiety linked to FoP can trigger a prolonged stress response further diminishing HRV [18], direct investigations examining the relationship between FoP and HRV remain scarce. Nonetheless, the interplay between FoP, pain perception, and autonomic regulation may be central to understanding the development and persistence of chronic pain.

The present study aims to examine the relationship between HRV, FoP, and perceived pain intensity. We hypothesise that both lower resting HRV and higher levels of FoP will be associated with increased pain ratings during cold stimulation. Furthermore, we predict that HRV will mediate the relationship between FoP and perceived pain intensity. Given the small sample size and cross-sectional design, the present analyses are intended to be exploratory and hypothesis-generating, with cautious interpretation of mediation results.

## 2. Materials and Methods

### 2.1. Ethical Approval

The study was approved by King’s College London Research Ethics Committee (HR-19/20-14149) and was conducted in accordance with the Declaration of Helsinki. Prior to participating in the study, all participants were provided with an Information Sheet. Written informed consent was obtained from each participant before the commencement of the experimental sessions, and they were informed of their right to withdraw from the study at any point.

### 2.2. Participants

Twenty-two healthy controls (HC) took part in this study. All participants were right-handed. Other inclusion criteria were: (1) aged between 18 and 65 years; (2) being able to understand English; and (3) being able to lie still for more than 1 h. Exclusion criteria included history of brain injuries, hypertension, neurological or psychiatric disease, and alcohol or drug abuse. Additional cardiovascular exclusion criteria were personal or family history of hypertension, smoking more than 5 cigarettes a day, BMI > 30, and history of orthostatic hypotension or carotid hypersensitivity. At the beginning of each visit, participants were tested for drug use (urine drug test) and alcohol consumption (alcohol breathalyser). To further exclude possible alterations of the ANS system, a Valsalva Manoeuvre was applied at the beginning of each session; a decrease in systolic pressure of >20 mm/Hg during the manoeuvre was defined as improper ANS functioning and specified as an exclusion criterion. Prior to each session, participants were required to abstain from alcohol for 24 h, non-steroidal anti-inflammatory drugs and paracetamol for 12 h, tobacco and nicotine containing products for 4 h and to limit caffeine intake to maximum of one caffeinated drink.

### 2.3. General Overview of the Experimental Session

Participants took part in one experimental session lasting approximately 1.5 h. One day prior to the session, participants completed a battery of questionnaires administered online via Qualtrics. At the start of the experimental session, baseline physiological activity was recorded for 5 min while participants rested in a supine position. This was followed by a 5 min cold pain induction, after which participants rated their perceived pain using a Visual Analogue Scale (VAS) (see below for further details). The remainder of the session involved a task measuring the attentional bias towards pain, the results of which are not reported in this publication.

### 2.4. Questionnaires

Anxiety was assessed by the State-Trait Anxiety Inventory [19]. Depression was assessed by means of the Center for Epidemiologic Studies Depression Scale (CES-D) [20]. Finally, the Fear of pain questionnaire–short form (FPQ-SF) was used to measure fear of pain. FPQ-SF is a revised version of the FPQ-III, reduced to 20 items and subdivided into four subscales: Fear of Minor Pain, Fear of Medical Pain, Fear of Severe Pain and the total score [21]. Pain catastrophising was assessed with the Pain Catastrophizing Scale (PCS) [22]. Sub-scores of pain rumination, magnification and hopelessness, as well as the total pain catastrophising score, were derived.

### 2.5. Pain Perception Measurements

Cold pain was applied using a locally developed aluminium probe (4 cm × 20 cm), which was placed on the volar surface of the left forearm. Cold water at 4 °C was continuously circulated through the probe via two chillers. Participants were instructed to rate the intensity of the cold pain on a VAS, with 0 indicating “no pain” and 100 representing “the maximum pain imaginable” for that specific condition. This set-up closely mirrored that used in our previous studies with healthy participants (from a different sample), where an association between cold pain and HRV was observed [23]. This stimulus induced a sustained experimental pain, which is deemed to persist long enough for psychological and coping factors to be activated [24]. Other studies demonstrate the utility of the cold pressor test in eliciting pain responses that are both consistent and relevant to clinical pain experiences [25].

### 2.6. Physiological Measurements

Continuous HR was monitored for 5 min using the CareTaker system (https://caretakermedical.net/) (accessed on 12 February 2026). Participants were instructed to lie still and relax during this period. Measurements were taken at rest, before any experimental procedures or pain-inducing tasks. The CareTaker cuff was placed on the ring fingers of the right hand to record HR continuously.

### 2.7. Statistical Analysis

All data are expressed as mean and standard deviation. Given the small sample size, non-parametric Spearman correlations were used for correlational analyses. Statistical significance was set at *p* < 0.05. All analyses were conducted using SPSS Version 23.0 for Windows (SPSS Inc., Chicago, IL, USA).

### 2.8. Pre-Processing of Physiological Data

Inter-beat intervals (IBIs) were determined for data collected using the CareTaker. Continuous non-invasive blood pressure and vital signs were acquired using the Caretaker Platform (Caretaker Medical LLC, Charlottesville, VA, USA; CT5 device). The IBI values underwent visual inspection, and any potential artifacts were manually removed. Subsequently, an artifact correction algorithm based on a threshold was applied to further refine the IBIs. This algorithm, adapted from the Kubios HRV Standard ver. 3.0.2 software (https://www.kubios.com/hrv-standard/) (accessed on 12 February 2026), compared each IBI interval to a local median calculated from the surrounding 5 heartbeats. If the IBI interval deviated beyond a specified threshold from the local median, it was identified as an artifact and replaced with the median value. Sequentially, different threshold values (0.45, 0.35, 0.25, 0.15, or 0.05 s) were employed, progressing from conservative to liberal, based on the severity of the individual artifact. A time-based HRV measure—the root mean square successive difference, RMSSD—was calculated. RMSSD is an HRV measure which is sensitive to changes in parasympathetic tone [26]. RMSSD values were log-transformed (natural logarithm), and logRMSSD was used in further analyses.

### 2.9. Mediation Analyses

Mediation analysis was performed to ascertain whether logRMSSD mediated the relationship between FoP and pain perception. Data analysis was performed with SPSS 22.0 for Windows (SPSS Inc., Chicago, IL, USA). First, Spearman’s coefficient was used to explore the association between logRMSSD and pain, FoP and pain, and logRMSSD and FoP. Next, three scores were entered in a mediation analysis using PROCESS (https://www.processmacro.org/index.html) (accessed on 12 February 2026) in IBM SPSS version 23.0 (IBM Corp., Armonk, NY, USA). FoP was introduced as an independent variable, cold pain was the outcome variable, and logRMSSD the mediator. The indirect effect was tested with 20,000 percentile-corrected bootstrap confidence intervals. Consistent with the exploratory, hypothesis-generating nature of the study and the limited sample size, we report 90% bootstrap confidence intervals to reduce the risk of Type II error while acknowledging the increased risk of Type I error; findings should therefore be interpreted cautiously and require replication.

## 3. Results

### 3.1. Participants

A total of 22 healthy, pain-free individuals participated in the experiment (mean age = 24.9 SD = 4.7, 11 females). All participants were free from any known history of chronic pain, psychiatric, or neurological conditions. None of the participants were taking any regular medications. All participants were university students.

### 3.2. Association Between FoP, Pain Catastrophising, Anxiety and Depression

Our correlational analyses did not reveal any significant associations between FoP, anxiety, symptoms of depression, or pain catastrophizing. A summary of the results can be found in Table 1.

### 3.3. Association Between Fear of Pain, Cold Pain Intensity and logRMSSD

We observed a pattern of associations involving the Fear of Severe Pain (FoSP) subscale, and both logRMSSD and cold pain rating. Specifically, FoSP was negatively correlated with logRMSSD (rho = −0.62, *p* = 0.006) and positively correlated with cold pain ratings (rho = 0.62, *p* = 0.008). LogRMSSD was also negatively correlated with cold pain ratings (rho = −0.59, *p* = 0.01). Taken together, these correlations indicate that higher FoSP is associated with lower resting HRV and higher cold pain ratings in this sample. No significant associations were observed for the FoP total score or the Fear of Minor Pain and Fear of Medical Pain subscales. We did not observe correlations between logRMSSD and STAI, CES-D, or PCS scores (all *p* > 0.05).

### 3.4. Mediation Analysis

We assessed whether resting logRMSSD statistically accounted for the association between Fear of Severe Pain (FoSP) and cold pain ratings using an exploratory mediation model. FoSP was significantly associated with lower resting logRMSSD (path a:b = −0.048, SE = 0.019, t = −2.58, *p* = 0.020), explaining approximately 29% of the variance in logRMSSD (R^2^ = 0.29). When FoSP and logRMSSD were entered simultaneously as predictors of cold pain ratings, the overall model was significant (R^2^ = 0.36, F(2, 15) = 4.16, *p* = 0.037). Within this model, neither the direct effect of FoSP on cold pain ratings (path c′: b = 2.41, SE = 1.59, *p* = 0.15) nor the association between logRMSSD and cold pain ratings (path b:b = −22.22, SE = 17.83, *p* = 0.23) reached statistical significance. The indirect effect of FoSP on cold pain ratings via resting logRMSSD was positive (ab = 1.07) and its 90% percentile bootstrap confidence interval did not include zero (90% CI [0.13, 2.40]). Consistent with the exploratory and hypothesis-generating nature of the study, this pattern of results is suggestive of an indirect association between FoSP and cold pain ratings via resting HRV; however, the absence of significant direct effects and the use of a 90% confidence interval indicate that this finding should be interpreted cautiously and does not establish temporal precedence or causality.

## 4. Discussion

This exploratory study examined associations between FoSP, cold pain ratings, and resting HRV in pain-free young adults. We observed that FoSP was associated with higher cold pain ratings and lower resting HRV (logRMSSD). In an exploratory mediation model, the pattern of findings was consistent with an indirect association between FoSP and cold pain ratings via resting HRV.

We replicate the well-established association between fear of pain and perceived pain intensity, consistent with prior meta-analytic evidence indicating a small-to-moderate relationship between these constructs [6]. The modest magnitude of this association reported in the literature suggests that a substantial proportion of variance in pain intensity remains unexplained, indicating that additional psychological and physiological factors may contribute to individual differences in pain perception. This provides a rationale for examining potential intermediary or contextual processes that may shape the fear–pain relationship.

In the present study, the association between fear of pain and pain intensity was specific to the Fear of Severe Pain (FoSP) subscale. In a sample of young, pain-free individuals, experimental cold pain may primarily engage fear responses linked to high-intensity or threatening pain, whereas fear of minor or medical pain may be less salient and show restricted variance. From this perspective, the specificity to FoSP is not unexpected and may reflect the relevance of threat-intensity appraisal in acute experimental pain contexts rather than a generalised fear of pain. Subscale-specific effects have also been reported in clinical settings. For example, in a case–control study of orofacial pain patients versus matched dental controls, McNeil and colleagues [27] observed that FoSP was selectively elevated in the pain group, whereas other fear-of-pain dimensions did not show the same pattern, and fear of pain was more closely related to congruent, pain-relevant fears (e.g., dental fear) than to broader indices of general psychological symptomatology. Such findings support the view that fear of severe pain may capture a threat-relevant dimension of pain-related fear that is particularly salient in contexts involving anticipated or experienced pain. At the same time, given the modest sample size and the exploratory nature of testing multiple FPQ-SF subscales, the possibility that the observed specificity reflects limited power, restricted variance, or chance findings arising from multiple comparisons cannot be excluded. Accordingly, this subscale-specific pattern should be considered hypothesis-generating and requires replication in larger samples with preregistered analyses and formal correction for multiple testing.

To further explore the mechanisms behind the association between FoP and pain perception, we investigated the role of the ANS, specifically focusing on HRV. HRV, as measured by RMSSD, is widely interpreted as an index of resting cardiac vagal tone. Our findings demonstrated that resting RMSSD statistically accounted for the association between fear of severe pain and pain intensity in this exploratory model, significantly enhancing our understanding of the mechanisms linking psychological factors with the subjective experience of pain. We have previously demonstrated that parasympathetic stimulation of the ANS can modulate fear [28,29] and pain [30]. In the case of pain, this modulation is disrupted in patients with chronic lower back pain [30]. These results support the hypothesis that the relationship between FoP and pain intensity may be better explained by mediating factors, particularly autonomic dysfunction, and more specifically, decreased vagal modulation.

Of note, our data partially diverge from two previous studies that investigated the effect of FoP on autonomic responses. Indeed, two previous studies found that FoP is unrelated to systolic blood pressure in response to pain in healthy controls [7,31]. This discrepancy may arise because HRV and BP are different measures of autonomic functioning. While both are linked to pain perception, HRV might be better suited to capturing the dynamic and flexible responses of the autonomic nervous system, especially in relation to psychological states like fear or anxiety, which involve greater vagal modulation [32].

Previous studies in fibromyalgia patients show that individuals with higher pain levels exhibit dysregulated autonomic responses to emotional stimuli (van Middendorp et al., 2013), and work in chronic pain samples indicates that psychological distress can statistically account for associations between HRV, baroreflex sensitivity, and experimental pain responses [33]. While these findings highlight the relevance of autonomic processes in established pain conditions, they leave unresolved whether autonomic alterations are a consequence of chronic pain or reflect pre-existing vulnerability factors that amplify pain responses. In this context, examining fear–autonomic–pain associations in healthy, pain-free individuals is particularly informative, as it allows these relationships to be studied in the absence of confounds related to chronic pain, long-term distress, or medication use. Our findings suggest that higher fear of severe pain, coupled with lower resting HRV, may represent a vulnerability profile that precedes pain chronification. Longitudinal studies following individuals from acute or sub-acute pain states will be critical to determine whether this pattern predicts the transition to chronic pain.

The observed pattern of associations among FoSP, resting HRV, and cold pain perception is consistent with the involvement of overlapping cortical and subcortical brain structures that support fear processing, autonomic control, and pain modulation. Our previous studies have shown that functional connectivity between the periaqueductal grey (PAG) and the ventromedial prefrontal cortex (PFC) was associated with both logLF-HRV (a HRV marker linked to the modulation of cardiac autonomic outflows by baroreflexes [34]) during cold-pain stimulation and participants’ cold pain ratings [35]. Furthermore, this connectivity was also linked to individual descending pain modulatory mechanisms [35], suggesting that the PAG-ventro-medial PFC network plays a crucial role in mediating the connection between pain perception and autonomic regulation via descending pain modulatory pathways. The PFC is integral in mediating the interaction between emotions and pain perception. Previous studies have shown that interventions such as Cognitive Behavioural Therapy lead to increased activations in areas of the PFC in patients with fibromyalgia. Functional connectivity between the right dorsolateral PFC and the dorsal posterior cingulate cortex has also been shown to inversely correlate with fear-avoidance beliefs in individuals with chronic pain [36]. These findings highlight the importance of the PFC in regulating emotional responses to pain, particularly in individuals with heightened pain-related fear.

There are several limitations to this study. First, the sample size is small, which may limit the generalizability of the findings. Additionally, the sample consisted of pain-free, healthy controls. While the lack of co-morbidities and medication use helps control for potential confounders, it is unclear whether these findings would be replicated in a chronic pain sample, where the psychological and physiological dynamics could differ. Mediation estimates can also be unstable in very small samples and sensitive to influential observations; although bootstrapping relaxes normality assumptions, it does not resolve small-sample instability. Accordingly, the indirect association reported here should be interpreted cautiously and replicated in larger, preregistered studies. Importantly, HRV was assessed exclusively at rest and prior to pain induction. As such, the present findings cannot be interpreted as evidence of altered autonomic regulation during pain itself. Instead, lower resting vagal tone may reflect a trait-like physiological context within which fear of severe pain is more strongly coupled to subjective pain experience. Future studies incorporating HRV and respiratory measures during pain induction will be necessary to determine whether pain-evoked autonomic responses further contribute to fear–pain coupling.

## 5. Conclusions

The present findings provide preliminary, hypothesis-generating evidence that fear of severe pain and resting HRV are associated with variability in experimental cold pain ratings in healthy controls. In an exploratory cross-sectional mediation model, resting HRV (logRMSSD) statistically accounted for the association between FoSP and cold pain ratings; however, the small sample size and cross-sectional design preclude causal inference, and effect estimates may be unstable. Future studies should test these associations in larger samples with preregistered analyses and longitudinal or experimental designs, ideally including autonomic measures collected during pain induction to evaluate whether pain-evoked autonomic responses better explain fear–pain coupling. Any potential translational implications should therefore be considered future research directions rather than clinical conclusions.

## Figures and Tables

**Table 1:** Spearman correlation coefficients (rho) examining associations between Fear of Pain Questionnaire–Short Form (FPQ-SF) scores, pain catastrophizing (PCS), depressive symptoms (CES-D), and trait anxiety (STAI-Trait) in healthy controls.

		CESD	STAI Trait	PCS	PCS-Rumin	PCS-Magnification	PCS-Helplessness
**FEAR OF MINOR PAIN**	rho	0.14	0.38	0.09	0.07	−0.06	0.21
	*p*	0.58	0.12	0.74	0.80	0.81	0.41
**FEAR OF MEDICAL PAIN**	rho	0.02	0.10	0.13	0.10	−0.10	0.25
	*p*	0.95	0.69	0.61	0.69	0.71	0.31
**FEAR OF SEVERE PAIN**	rho	−0.16	0.11	0.01	0.09	−0.10	0.05
	*p*	0.53	0.66	0.98	0.71	0.69	0.84
**FEAR OF PAIN-Total Score**	rho	0.05	0.28	0.10	0.12	−0.12	0.22
	*p*	0.84	0.26	0.71	0.63	0.64	0.38

## Data Availability

The data supporting the findings of this study are available from the corresponding author upon reasonable request.

## Abbreviations

The following abbreviations are used in this manuscript:

ANS	Autonomic Nervous System
BP	Blood Pressure
CBT	Cognitive Behavioural Therapy
CES-D	Center for Epidemiologic Studies Depression Scale
FPQ-SF	Fear of Pain Questionnaire–Short Form
FoP	Fear of Pain
FoSP	Fear of Severe Pain
HC	Healthy Controls
HR	Heart Rate
HRV	Heart Rate Variability
IBI	Inter-beat Interval
LF	Low Frequency
PAG	Periaqueductal Grey
PCS	Pain Catastrophizing Scale
PFC	Prefrontal Cortex
RMSSD	Root Mean Square of Successive Differences
STAI	State-Trait Anxiety Inventory
VAS	Visual Analogue Scale
